# Tensile Forces Originating from Cancer Spheroids Facilitate Tumor Invasion

**DOI:** 10.1371/journal.pone.0156442

**Published:** 2016-06-07

**Authors:** Katarzyna S. Kopanska, Yara Alcheikh, Ralitza Staneva, Danijela Vignjevic, Timo Betz

**Affiliations:** 1 Institut Curie, PSL Research University, CNRS, UMR 168, F-75005, Paris, France; 2 Sorbonne Universités, UPMC Univ Paris 06, CNRS, UMR 168, F-75005, Paris, France; 3 Centre National de la Recherche Scientifique, UMR168, 25 rue d'Ulm, 75248 Paris cedex 05, France; 4 Zurich University of Applied Sciences, Institute of Chemistry and Biotechnology, Einsiedlerstrasse 31, 8820 Wädenswil, Switzerland; 5 Institute Curie, PSL Research University, CNRS, UMR 144, F-75005, Paris, France; 6 Institute for Cell Biology, Center for Molecular Biology of Inflammation, Münster University, Münster, Germany; University of California, San Diego, UNITED STATES

## Abstract

The mechanical properties of tumors and the tumor environment provide important information for the progression and characterization of cancer. Tumors are surrounded by an extracellular matrix (ECM) dominated by collagen I. The geometrical and mechanical properties of the ECM play an important role for the initial step in the formation of metastasis, presented by the migration of malignant cells towards new settlements as well as the vascular and lymphatic system. The extent of this cell invasion into the ECM is a key medical marker for cancer prognosis. *In vivo* studies reveal an increased stiffness and different architecture of tumor tissue when compared to its healthy counterparts. The observed parallel collagen organization on the tumor border and radial arrangement at the invasion zone has raised the question about the mechanisms organizing these structures. Here we study the effect of contractile forces originated from model tumor spheroids embedded in a biomimetic collagen I matrix. We show that contractile forces act immediately after seeding and deform the ECM, thus leading to tensile radial forces within the matrix. Relaxation of this tension via cutting the collagen does reduce invasion, showing a mechanical relation between the tensile state of the ECM and invasion. In turn, these results suggest that tensile forces in the ECM facilitate invasion. Furthermore, simultaneous contraction of the ECM and tumor growth leads to the condensation and reorientation of the collagen at the spheroid’s surface. We propose a tension-based model to explain the collagen organization and the onset of invasion by forces originating from the tumor.

## Introduction

Metastasis is a major cause of death for cancer patients and the end result of a multistep process that involves local tumor invasion, the dissemination of tumor cells to distant organs and an adaptation to various tissues [1]. The mechanisms of invasion have been widely studied in the past [2]. Invading cells often display characteristic Epithelial to Mesenchymal Transition (EMT) markers, such as down- regulation of E-cadherin and upregulation of vimentin, and lose some epithelial characteristics, such as apical- basal polarity [3].

Tumor microenvironment is characterized by distinctive mechanical properties as compared to healthy tissues. Extracellular matrix (ECM), mainly composed of collagen [4], accumulates in tumor stroma and it is responsible for the stiffness increase observed in many tumors [5]. Tumor progression is also accompanied by a distinct collagen architectures [6], termed tumor-associated collagen signatures (TACS), that have been correlated to patient prognosis. Initially, there is an increase in collagen amounts in the surrounding tissue (TACS-1). In the later states collagen fibers become aligned parallel to the tumor surface (TACS-2) [4–6]. Finally, in invasive tumors, collagen fibers are found to be aligned perpendicular to the tumor boundary (TACS-3), which also correlates with the direction of cellular invasion [7]. TACS have been described as a prognostic marker for patient’s survival [5]. Similarly, a strong correlation between metastatic potency and intra-tumoral matrix alignment, including radial and parallel alignment of collagen fibers has been described in colorectal cancer mouse model [8]. A positive feedback between the tumor mediated changes in the collagen and the cancer as well as cancer associated cell types has been suggested [9], which may explain the stable and reproducible occurrence of these collagen structures.

The modifications of the tumor stroma is known to be a result of biochemical/enzymatic processes, where cancer cells as well as cancer associated fibroblasts (CAFs) play a key role in degradation and remodeling of the matrix [8,10–12]. This biochemical remodeling has been extensively studied and depends on degradation by matrix metalloproteinases (MMPs) and ECM stiffening by lysyl oxidase (LOX) [10]. The stiffening of the matrix was suggested to be a driving factor for invasion [13], however more recent studies identify the matrix pore size rather than rigidity as the critical property modulating cancer cell invasion [14,15]. Here we address the question to which extent a pure mechanical remodeling of tumor ECM may be generated by the forces applied by the tumor cells on the ECM. Such pulling forces on the ECM that are created either by the cancer cells themselves, or by cancer associated fibroblasts (CAFs) are known to contribute to the matrix stiffening and the fiber alignment around the tumor [16–23].

Due to the spatial complexity of the tumor’s 3D environment, regulation and outcome of traction forces on collagen are difficult to study *in vivo*. However, recent advances in developing reliable 3D *in vitro* cancer models, allow dissection of a mechanical remodeling process. Especially, multicellular cancer spheroids embedded in the ECM have been found useful to study invasion [24,25]. Studies using these 3D spheroid models showed that cells actively change the surrounding matrix by mechanical alignment and strain stiffening [26]. In brain tumor spheroid systems, both compressive pressure and tension on the matrix microenvironment has been shown to be linked to the tumor’s invasive dynamics [27]. The detailed dynamics and correlation between the mechanical remodeling and invasion has not yet been studied. Additionally, studies showed that mechanical pressure and ECM elasticity regulate growth of spheroids [28,29].

Previous studies showed that single cancer cells exert traction forces on fibrillar matrix proteins that lead to ECM deformation [12]. Analysis of the collagen fiber alignment *in vivo* and *in vitro* had suggested that forces originating from the tumor spheroid are important for this alignment [7,20,21,23]. While contractile forces have been observed in the single cell context to rearrange collagen fibers [12,30], the growth of a tumor rather suggests pushing of the ECM at the tumor surface, which effectively compresses the collagen matrix. Therefore, two contributions of compression can be isolated, a pushing force due to tumor growth and a pulling force that is applied by the cancer cells on the ECM. On the tumor surface collagen may buckle, but also break or be degraded. The effect of contractility in other cell systems such as fibroblasts has been well studied, where it was shown that collagen contraction creates patterns of tension and fibers’ alignment, which facilitate cell migration [31–33]. The importance of tension in cell migration is also provided by studies on culturing fibroblasts in floating, low tension 3D gels as well as anchored (restrained), high tension gels [34,35].

The main aim of the work presented in this paper is to study the effect of the tumor-mediated ECM’s contraction and to show that tension is generated and affects cell invasion.

## Results

### Three-dimensional model of cancer cell invasion

To study the invasion of cancer cells in the ECM we used a previously described 3D collagen invasion assay that was adapted to our experiments (S1A and S1B Fig) [25,36]. Spheroids derived from the murine colon carcinoma cell line CT26 [37], transfected to stably express cytoplasmic GFP, are recorded by spinning disk microscopy to visualize invasion. CT26 cells are characterized by a mesenchymal phenotype, especially by lack of E-cadherin expression [37]. Therefore they have a very high invasive potential and as they encounter a favorable environment (the ECM) they migrate out of the spheroid. Multicellular spheroids were generated using a classical agarose cushion protocol and embedded in collagen type I attached to the surface of the dish (S1B Fig). Collagen gel was polymerized at room temperature (≈22°C) to provide matrix architecture comparable to *in vivo*. Geraldo et al. showed that collagen polymerized *in vitro* in room temperature resembles fibers found *in vivo* in animal models with a network consisting of a mixture of a few thin bundles and many thick bundles varying from 0.72 up to 1.56 μm thickness. In contrast, collagen polymerized in 37°C didn’t show similarities to animal tissue [38]. In our study the physiologically more relevant polymerization condition at room temperature was chosen (S1C Fig and S1 Movie) [25]. This is furthermore supported by *in vivo* study in mouse colon cancer, where we observe the organization similar to the reconstituted system used here.

**Fig 1:**
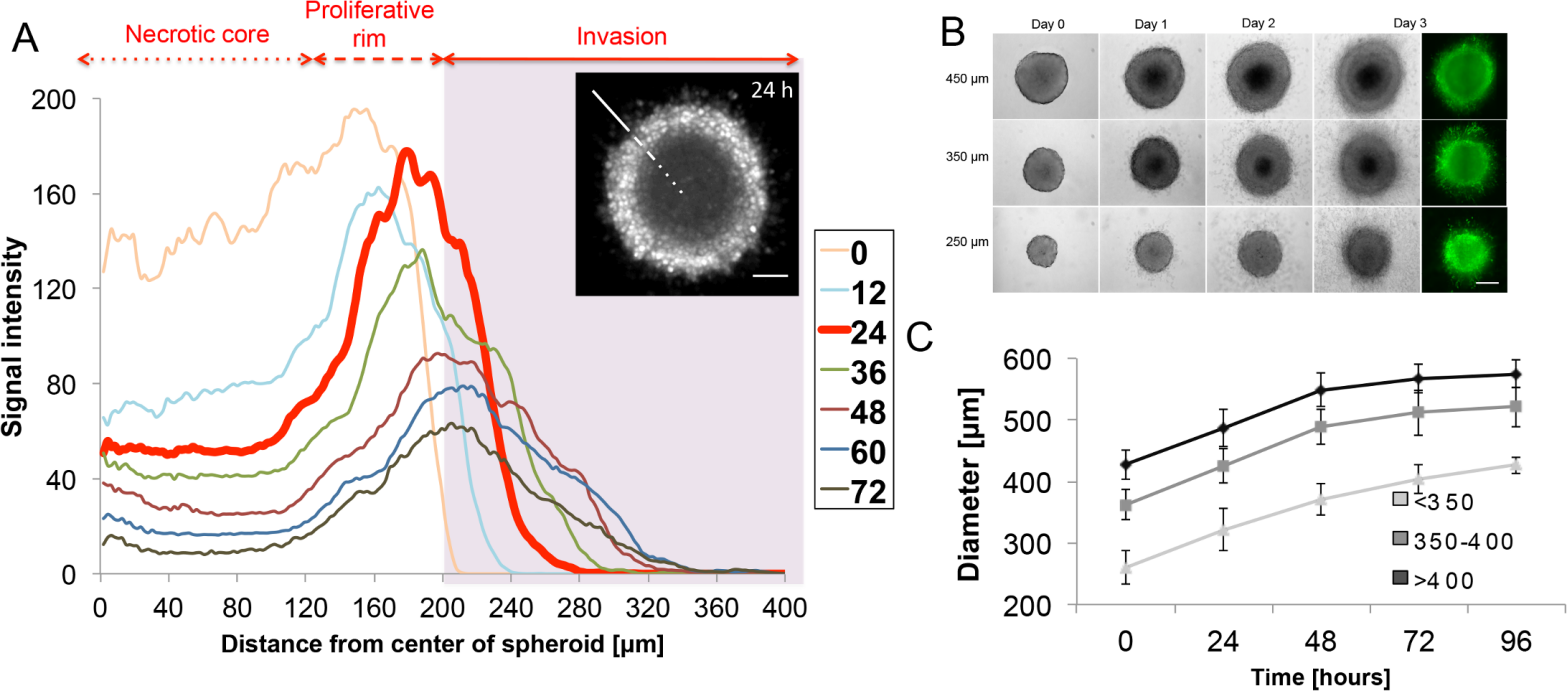
CT26 cell line spheroids characterization. **(A)** Radial profile of spheroid’s GFP fluorescence over 72 hours of invasion into collagen type I. Different zones of the spheroid can be distinguished: dark area, previously described as necrotic core (dotted line), proliferative rim (dashed line) and invasion (line and shaded area). Insert: representative image of spheroid (GFP) 24 hours post-seeding. Scale bar: 100 μm. **(B)** Morphology change over time of different-sized spheroids seeded into in collagen type I. Spheroids were imaged in bright field and fluorescence (GFP). Scale bar: 200 μm. **(C)** Growth kinetics of spheroids for three different initial sizes (<300 μm, 350–400 μm and >400 μm). Values are mean ± standard deviation of n = 6–12 from three independent experiments.

Fig 1A shows representative confocal fluorescence images (3D projection) of a GFP-CT26 spheroid, where cells disseminate from the spheroid in the ECM, hence mimicking invasion.

Spheroids increase in size (Fig 1B) as a result of cell proliferation. As shown by Alessandri et al. [36], the core of CT26 spheroids is necrotic, similar to other spheroid systems. In contrast, cells at the rim detach and invade into the collagen. In radial profiles of the GFP fluorescence the time dependent development of different zones can be observed. At t = 0h (Fig 1A, yellow) the fluorescence in the inner spheroid is only slightly smaller than at the border. However, after 24h three zones are apparent, (Fig 1A, red): the necrotic core (the dark area inside the spheroid, low signal intensity), the proliferative rim (signal intensity peaks), and invasion zone (shaded area). These features mimic the morphology of *in vivo* tumors [24].

To choose the optimal initial size of spheroids that would contain three distinct zones, we analyzed growth (Fig 1C) and invasion of spheroids of three different diameters, namely 450 μm, 350 μm and 250 μm (Fig 1B). The dark area in smaller spheroids (250 μm) becomes visible after approximately three days when the spheroid has reached 300 μm in diameter (Fig 1B), while bigger spheroids develop the core already after one day of culturing in collagen. To test if the initial size of spheroids affects the invasion, we quantify the invasion area for spheroids of different initial diameters and find no significance dependence of the relative invasion area on the initial spheroid size (p-value > 0.05) (data not shown). As the small spheroids show continuous growth and provide easiest access for 3D imaging, we exclusively use spheroids between 300–350 μm in initial size for this study.

### Cancer cells contract collagen network

**Fig 2:**
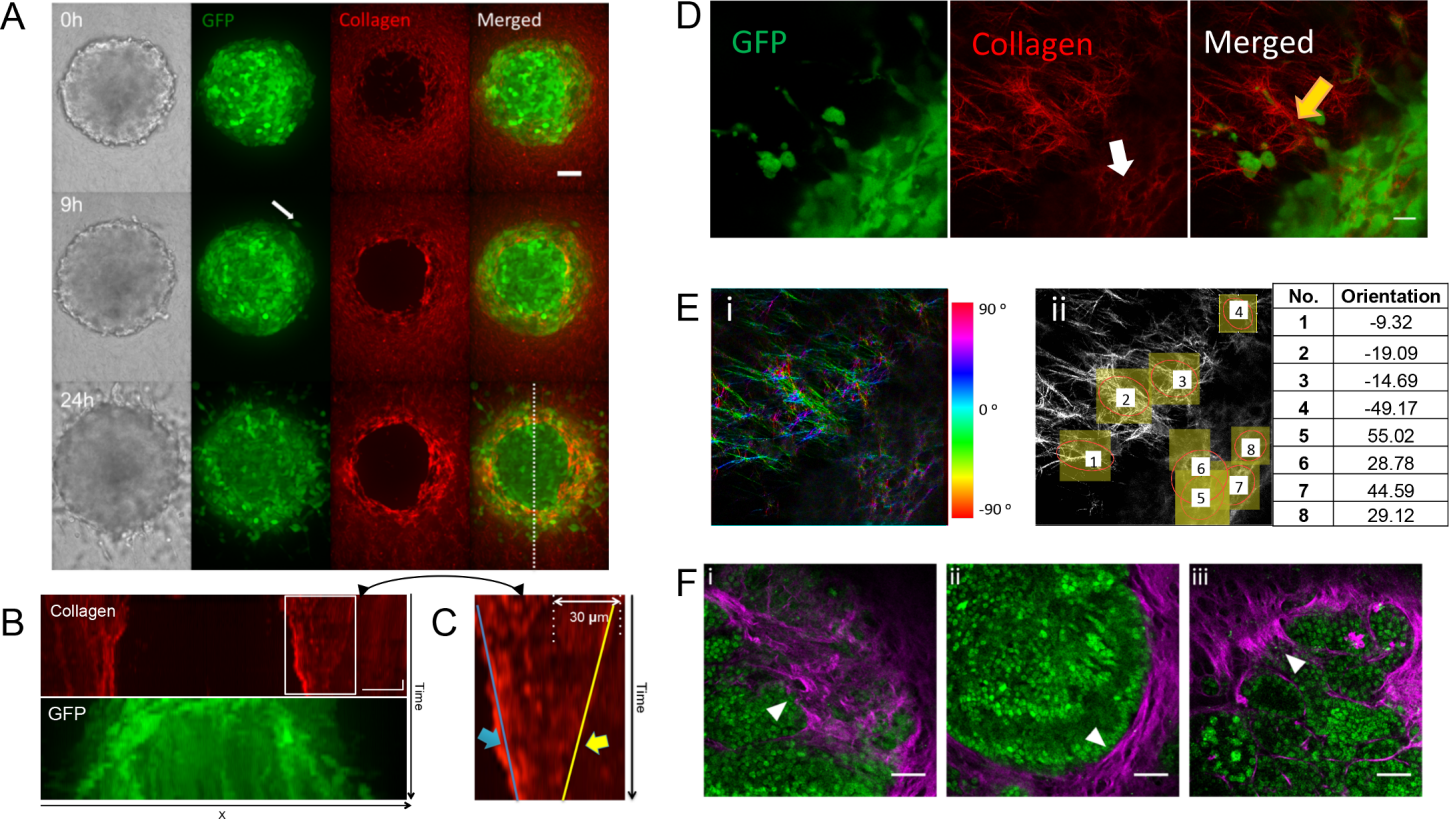
Invasion of cancer cells CT26 into collagen type I. **(A)** Image sequence of CT26-GFP (green) cell invasion in TAMRA-labeled collagen type I (red). Cells initiate invasion (white arrow) after approximately 9 hours (onset of invasion). Scale bar: 50 μm. **(B)** Kymographs of collagen and cells from the image sequence (S2 Movie). Images were taken every one hour for 24 hours. Scale 50 μm and 3 hours. **(C)** Magnification of the boxed region showing movement of the collagen fibers towards the spheroid. The kymograph illustrates the two antagonizing movements of compression due to spheroid growth (close to spheroid, blue arrow and blue line), and the collagen contraction in the invasion zone (stripes toward the spheroid, yellow arrow and yellow line). **(D)** Zoom in confocal images of CT26-GFP multicellular cancer cell spheroid with cells invading (green) into collagen type I network (red) showing the collagen organization: parallel fibers (white arrow) and radial fibers (yellow arrow). Scale 20 μm. **(E)** Collagen fiber orientation i) Color-coded orientation map. ii) Quantitative orientation measurement (table—angles) on selected ROIs (circles). Spheroid surface orientation: 45°, orientation normal to surface: -45° **(F)** Collagen signatures found in a single NICD/p53^-/-^ mouse intestinal tumor are imaged using intravital two-photon microscopy. i) TACS-1, curly collagen structure; ii) TACS-2, straight and aligned collagen, parallel to the tumor edge and iii) TACS-3, collagen aligned perpendicularly to the tumor edge. Epithelial cancer cells (nuclear GFP, green), collagen (SHG, magenta). Arrowheads point to distinct collagen organization. Scale bars, 50μm.

To study the dynamic rearrangement of the collagen matrix during invasion we performed live imaging with a time resolution of 1h over 24 hours (S2 Movie). The spheroid’s initial size increases over time (Fig 2A, bright field and GFP) and at 9 hours first cells are observed to leave the spheroid (onset of invasion) (Fig 2A white arrow). The distribution of invading cells at 24 hours is uniform around the spheroid.

As collagen is polymerizing in presence of the spheroid, we can observe penetration of the gel into first cell layers (Fig 2A and 0h). Additionally, the images represent maximum projections of 40μm thick z-stacks, which would also lead to a certain overlay. Over time, the collagen organization changes as a function of distance to the spheroid. Namely, collagen fibers are aligned parallel to the edge of spheroid at close proximity (white arrow Fig 2D) and appear radially aligned further away from the surface (yellow arrow Fig 2D). This alignment was quantified by measuring the angular orientation at different positions (Fig 2E). It should be noted that only few cells have reached the region with alignment perpendicular to the surface. This excludes that the realignment is due to massive reorganization of the collagen by tumor cells, for example by the creation of voids in the collagen during migration. Our finding of this variable arrangement confirms similar *in vivo* and *in vitro* reports [6,39]. The time dependent organization shown in Fig 2A allows monitoring collagen changes during spheroid’s growth and invasion. We observe (Fig 2A) that collagen is remodeled over 24h, changing from an isotropic organization at time 0h to the described parallel alignment on the surface and that appearance of radial bundles in the surrounding ECM. Possible parallel alignment of fibers during the collagen polymerization phase is not observed, but might be beyond the resolution of the current data. Blurred areas that are observed around the spheroid might be associated to the enzymatic degradation by cells or cells in different z positions. Previous experiments have shown that the pushing forces of the growing spheroid alone can already lead to the parallel alignment at the spheroid surface [40].

The kymograph (Fig 2B and 2C) shows a rich pushing/pulling behavior. While the collagen close to the spheroid surface is pushed outward by the spheroid growth, 30 μm away from this pushing zone we observe a pulling of the collagen towards the spheroid, resulting in an overall contraction of the collagen ECM around the spheroid. The kymograph (Fig 2C) illustrates the two antagonizing movements of compression due to spheroid growth (white arrow), and the collagen contraction in the invasion zone (yellow arrow). This somewhat contradictory behavior of simultaneous pushing (due to spheroid growth, blue line, Fig 2C) and pulling (by cell forces, yellow line, Fig 2C) creates a compression zone at the spheroid surface where the collagen is rearranged tangential to the spheroid (Fig 2). Furthermore, the pulling forces deform the collagen radially, thus inherently creating a preferential alignment of the fibers perpendicular to the spheroid surface (Fig 2D) that is very similar to the fiber alignment observed *in vivo* (Fig 2F). Such behavior is also known from sheared collagen fibers *in vitro* [30].

### Cell-induced collagen contraction precedes invasion

**Fig 3:**
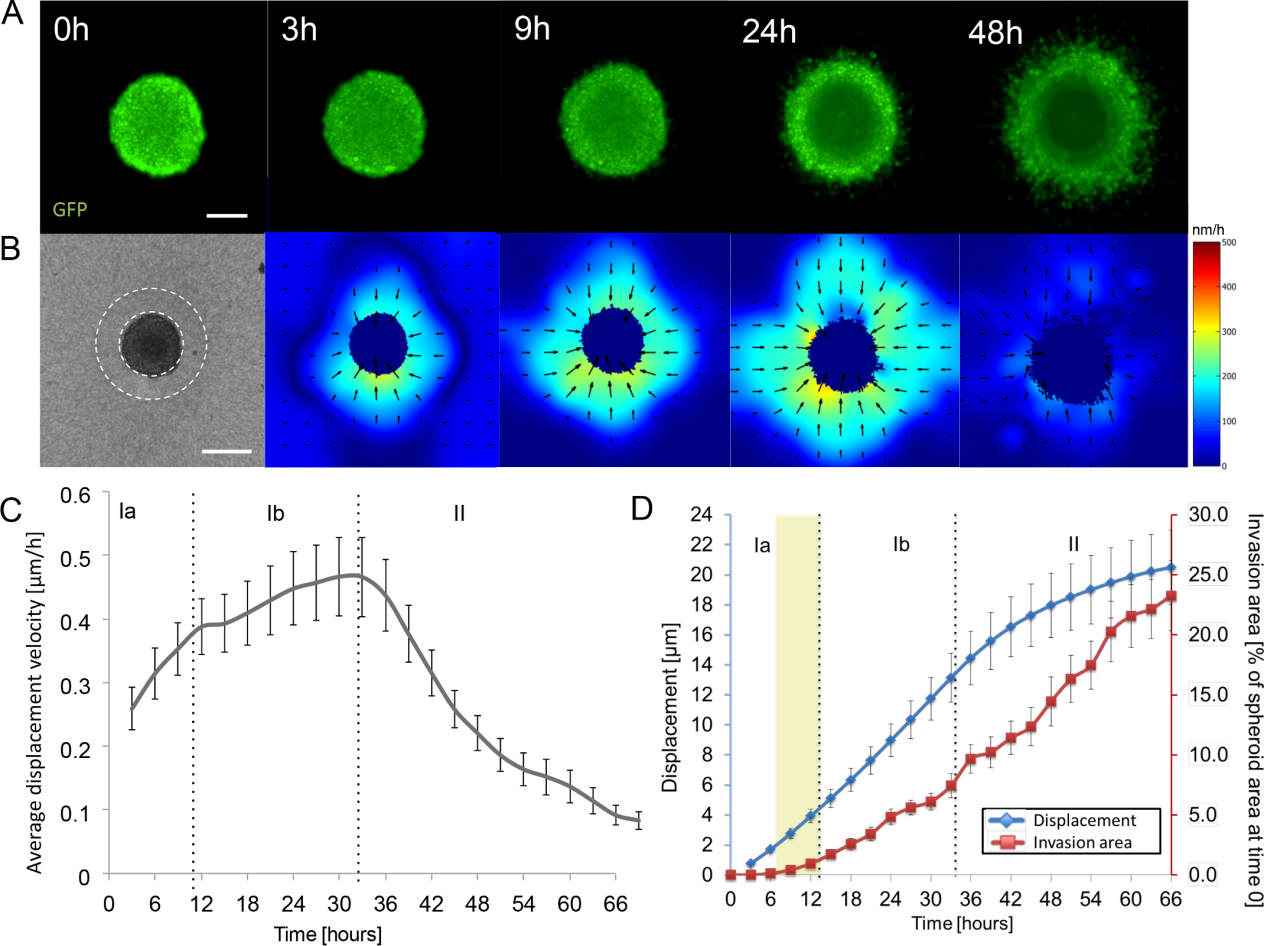
Quantification of collagen contraction and invasion of GFP-positive CT26 cells. **(A)** Selection of images showing cell invasion (green) in collagen over 48 hours. At approximately 9–12 hours cells start to leave the spheroid (onset of invasion). **(B)** Initial bright field image of spheroid in collagen at time 0 hours and velocity maps of collagen contraction. Arrows indicate the directionality of contraction and color code refers to speed of contraction (red-high and blue-low). The dark blue fields mask movement of cells and growth of spheroid, which is not taken into account when calculating the displacement velocity map. For quantification average displacement velocity was calculated from the 100 μm area outside the spheroid indicated by the dashed lines. **(C)** The speed of collagen contraction (μm/h) over a period of 66 hours. Different phases can be distinguished for contraction speed: Ia- initiation with increased acceleration, Ib- reduced acceleration and II–slowdown, separated by the dotted line. Invasion onset is indicated with yellow shading. **(D)** Blue line shows the cumulative of the average collagen deformation (of Fig 3C) and invasion area (red). The invasion area was normalized for each sample to the initial spheroid area. Scale bar: 150 μm (bright field and GFP) and 300 μm (velocity map). All values are mean ± standard error (n = 19) from five independent experiments.

The kymograph-based inspection suggests that collagen contraction starts immediately after seeding the spheroid in the ECM, while the cells invade only later. To study the timing of contraction and invasion we quantify its time dependence. Invasion is measured based on fluorescent images of the GFP positive CT26 cells (Fig 3A), by quantifying the area of cells that appear detached from the spheroid and normalizing it with the area covered by the spheroid (S2 Fig). The collagen contraction detection (Fig 3B) is based on a previously described correlation algorithm applied to bright field images [41], which is validated by tracking fluorescent beads embedded in the collagen gel (S3–S5, S8 and S9 Figs).

The resulting velocity map sequence (Fig 3B) visualizes the contractile flow speed as color-code, while the arrows give direction of the flow. Already 3 hours after seeding, we detect a contractile collagen flow (light blue to green shading) towards the spheroid (black arrows). The contraction decreases radially and shows some tangential variations. The contraction zone around the spheroid expands over time and contraction speed typically peaks at about 30h post-seeding, followed by a gradual decrease.

The contraction is quantified by calculating the mean contraction speed in an area of 100 μm around the spheroid’s surface for each frame (Fig 3B white circle). Fig 3C shows the resulting time dependent contraction velocity averaged over several different spheroids (n = 19). The overall reproducible properties suggest that the spheroid model system can be used to study variation of invasion and mechanical interactions between the spheroid and the model ECM.

Three phases can be distinguished phenomenologically (Fig 3C). In the first phase (Ia) contraction is initiated immediately after sample preparation with a well-defined contraction speed. The contraction accelerates with a linear speed increase until 12h post-seeding. The second phase (Ib) starts with a change in speed increase and lasts until about 30h, when the contraction speed peaks. In certain cases, the contraction even plateaus to a stable, lower velocity during the second phase (see S3 Fig). After reaching the contraction velocity peak at about 30 to 33 hours we observe a general deceleration of the contraction down to an almost growth stop with a final speed of only 0.1+- 0.02 μm/h at the end of the recoding (66 h) (phase II). By comparing time dependence of total collagen deformation with invasion area (Fig 3D) we found that the contraction precedes invasion in average by about 9-12h, which confirms the results of the kymograph in Fig 2. The onset of invasion appears only after the average collagen deformation reaches 2–4 μm (Fig 3D). Interestingly, at the beginning of phase II (33h), we observe a mild acceleration of invasion (Fig 3D) occurring at the time the contraction speed peaks. Furthermore, these results show that the invasion area continues to increase in phase II, although the collagen contraction slows down (phase II), suggesting that it is not the collagen movement that influences the invasion.

### Collagen contraction creates tension in the network

Collagen contraction suggests that the cells on the outer layer of the spheroid pull on the fibers. We wanted to determine if the collagen movement was a result of cells’ active traction forces or simply caused by viscous flow due to collagen digestion at the spheroid surface. The importance of traction was evident when seeding two spheroids in close proximity. In this setup we observe that cells migrate predominantly across the area between spheroids. This migration patterns also corresponds to the pattern of the expected tensile forces (S4 Fig), as was observed in other model systems [31–33,35,42]. In such multi-spheroid experiments, the observed radial alignment is typically more pronounced than in single spheroid analysis.

**Fig 4:**
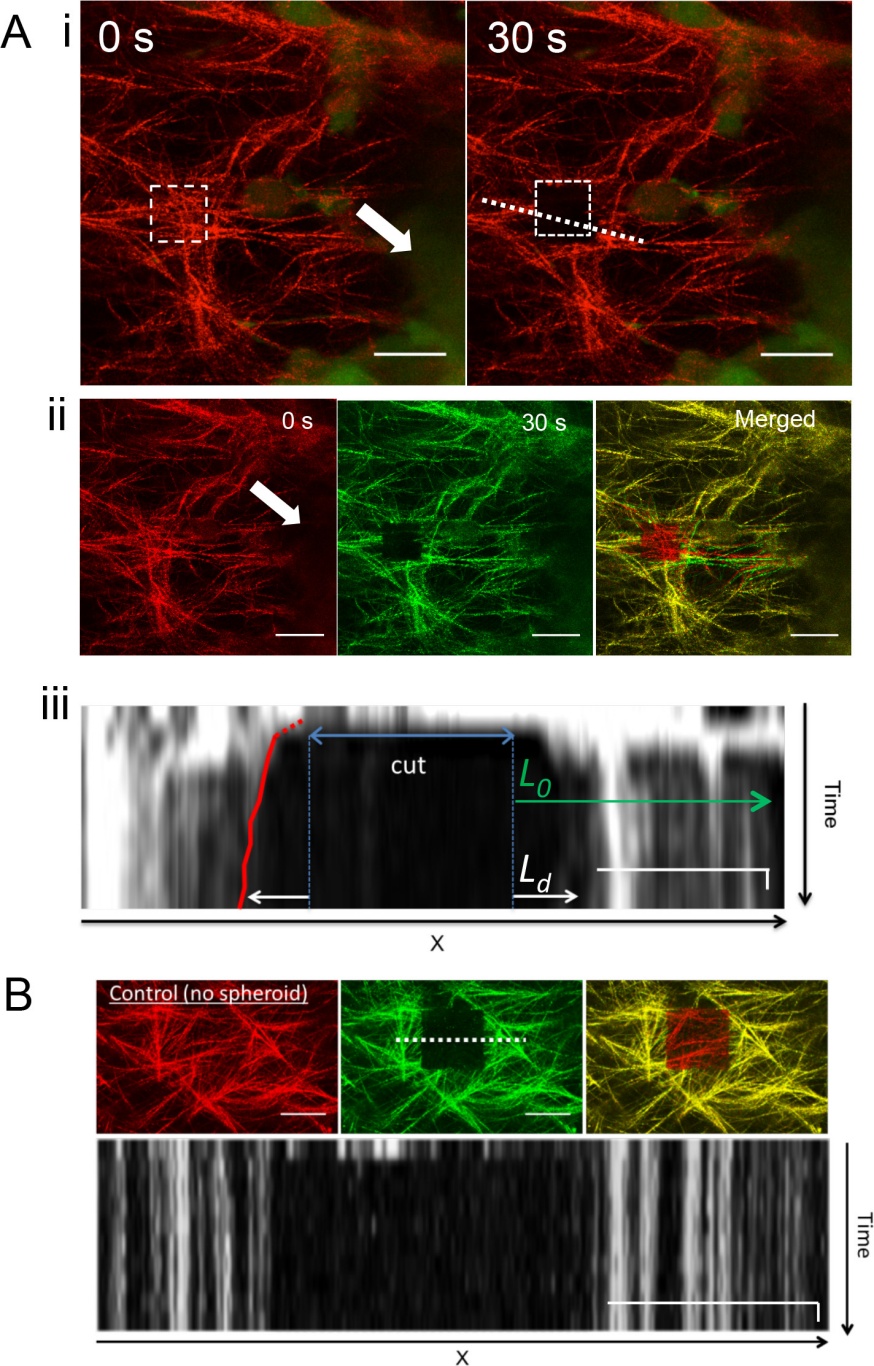
Ablation of spheroid contracted collagen fibers. **(A)** To study the presence of tension in the moment when contraction has almost stopped, collagen fibers were cut 48 hours post-seeding of a CT26 GFP spheroid in collagen: (i) Representative images of ablation area at 0 and 30 second after cutting. White arrow indicates the position of the spheroid. The square gives the ablated area and the dashed line represents the line chosen for the kymograph in iii. (ii) Co-localization of collagen fibers before and 30 seconds after ablation performed next to the spheroid (white arrow). Red and green color is applied to reflection fluorescence before and after the cut respectively visualize relocation of collagen fibers. Scale bar: 20 μm. (iii) Kymograph of frame sequence from S6 Movie. The red line traces the fiber retraction. The strain is defined as u = L_d_ /L_0_, and is in the example presented 0.23. Scale bar: 15 μm (long bar) and 10 seconds (short bar). **(B)** Control experiment in collagen without spheroid. No tension is observed, as there are no signs of retraction after the cut. Scale bar: 15 μm (long bar) and 10 seconds (short bar).

To estimate the elastic tensional forces within the collagen we ablated collagen 48h post-seeding using an 800 nm pulsed laser (Fig 4A and S6 Movie). At this time-point the collagen flow has largely stopped, which means that the systems has either relaxed any mechanical tension by viscous flow or that a mechanical equilibrium is established where pulling forces from the cells are balanced by mechanical tension in the collagen. Collagen is imaged before and after ablation with a time-resolution of 10 sec. Successful ablation is confirmed by reflection microcopy on the same setup, which does not depend on a fluorescence signal, but the presence of the collagen fibers.

We observe an initial rapid fiber displacement right after cutting (Fig 4A ii and S6 Movie), followed by a continuous decaying relaxation process. Fibers move to opposite directions as indicated on the kymograph (Fig 4A iii). Control gels without spheroids show no sign of tension and relaxation (Fig 4B). The observed movement directly shows that the network is at this time-point still under mechanical tension. This suggests that the contraction is caused by forces generated by the spheroid. Furthermore, the fact that the retraction after the cut is aligned in the direction of the spheroid shows that the cells on the spheroid pull on the collagen, which directly explains the observed collagen flows. Strain (Fig 4A iii) was calculated by dividing the deformation length *L*_*d*_ at the cut (in μm) by the distance from the cut at which no movement of the collagen was measured after the cut also in μm (*L*_*0*_). The average strain after the cut was found to be u = 11+- 5.3% (mean +- SEM n = 14).

### Invasion is locally suppressed in the absence of tension

**Fig 5:**
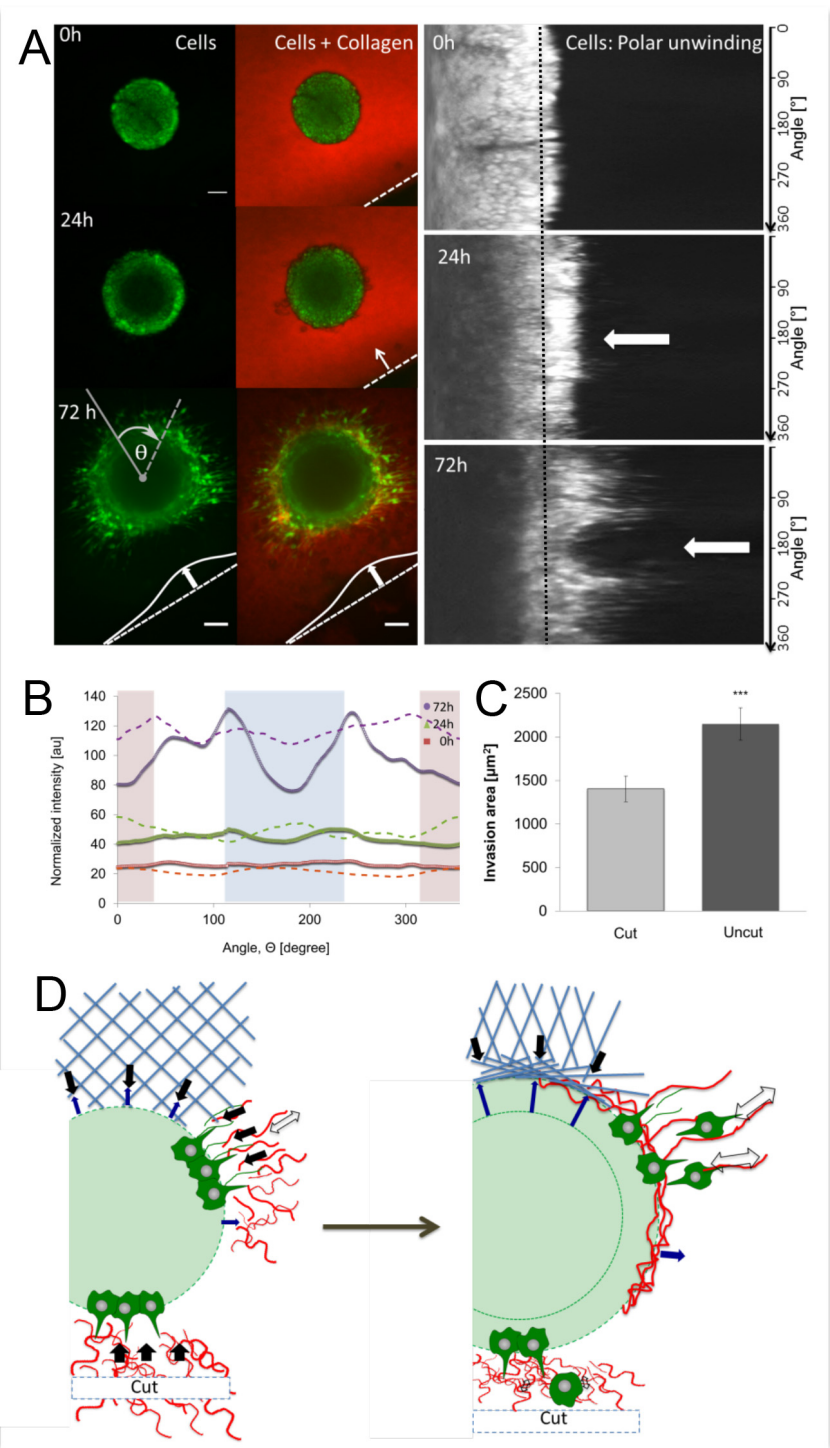
CT26 invasion after cutting the collagen gel close to the spheroid. **(A)** Collagen (z projection, 4x objective): red TAMRA collagen was polymerized and cut with scalpel on one side. Left images show the invasion of GFP-positive cells (green) at 0, 24 and 72 hours post-seeding. The dashed line indicates an edge of the cut collagen and the arrow shows the direction of collagen contraction. Right: Fluorescent images of angular unwinded spheroid where each horizontal line corresponds to the radial fluorescence intensity profile. The y-axis of these images corresponds to the different angles. Arrows show the area of invasion on the side of the cut. The black dotted line makes the left limit of the area used to calculate the average fluorescence as shown in B. **(B)** Average fluorescent intensity of cells extending over the initial spheroid surface as a function of angle Θ. This is used to estimate the outgrowth of cells from the original spheroid size. The different colors represent the average intensity after 0 (red), 24 (green) and 72 hours (violet) of invasion. The shading of the graph shows the side facing the cut (light blue), the opposing side facing the inside of the gel (light red) and the two sides perpendicular to the cut (white area). The dotted line represents the outgrowth in spheroids embedded in uncut collagen at the corresponding times. **(C)** Invasion area quantification on the side of the cut (after 0 hours) and average invasion area on uncut side. Invasion area was quantified as in Fig 3 (mean ± standard error, n = 54 of 6 independent experiments). **(D)** Sketch of tension-dependent invasion model.

To determine a possible relation between the tensional forces applied by the spheroid to the collagen and cancer cell invasion, we asymmetrically relaxed tension by macrosurgery experiments. This was done by a series of cut experiments on collagen gels with embedded spheroids, where the gels are asymmetrically cut on one side of the spheroid (S5 Fig) right after collagen is polymerized (Fig 5A). During collagen contraction, the built-up of mechanical tension in the direction facing the cut is suppressed due to the free boundary. Invasion and contraction is monitored over 72 hours (S7 Movie) and an asymmetry of invasion can be observed (Fig 5A, 72h). While on the side of the cut the collagen still contracts, we observe an inhibition of cell invasion on both, the side facing the cut (light blue shaded area Fig 5B) and the opposing side facing the inside of the gel (light red shaded area Fig 5B). However, on the two sides perpendicular to the cut, invasion is facilitated (white area Fig 5B). This was quantified by measuring the average fluorescence intensity from the cells outside the spheroid as a function of angle. While at 0h, no strong deviation of the fluorescence becomes evident, already after 24h there is a slight increase at the edges of the cut area, which becomes very dominant after 72h as quantified by the of two peaks in Fig 5B. As the contractile forces are not balanced in the direction of the cut, we observe an overall movement of the spheroid away from the cut, which in turn reduced the tension built up on side opposing the cut. Interestingly, we also see a decrease of invasion in the direction opposing the cut (Fig 5B light red). The decrease of invasion is quantified by comparing the invasion in the direction of the cut and the rest of the spheroid (Fig 5C, n = 54 spheroids).

## Discussion

Similar to most motile cells, cancer cells apply physical forces on their surrounding matrix [9,43] and dynamically remodel the ECM. Both characteristics suggest that they can actively change their environment and these changes may improve the conditions for cancer cell invasion [16].

While the effects between migrating cells and the ECM have already been studied previously on the single cell level, it should be pointed out that studies on the spheroid level allow to gain insights into possible collective effects. The large body on single cell work is used to understand force and tension elements in such mechanically isolated systems. Single cells have been shown to apply contractile forces and tension on the collagen that result in both mechanical and biochemical remodeling, similar to the spheroid. However, due to the limited forces of single cells the effects on the global ECM architecture remain small compared to the effect of cancer spheroids. Exceptions to this are free-floating, low-tension gels where the matrix is not able to resist the traction forces of individual fibroblasts. In these free systems the ECM simply contracts and both fibroblast migration as well as differentiation of fibroblast into myofibroblasts are inhibited. In contrast, tension loaded anchored (restrained) gels allow the generation of contraction forces and the migration into the matrix by single fibroblasts is supported [44]. This suggests that fibroblast migration depends on the ability to build up internal tension and raises the question whether cancer cell invasion may actually also depend on tension in the ECM.

Additionally to the tension generation *in vivo* tumors (Fig 2F) can be characterized by well described collagen signatures [7], which can be mimicked *in vitro* by embedding cancer spheroids in a ECM. This striking effect of cancer spheroids on the ECM structure hints for a collective effect that should be discussed differently than the well known single cell contractions [23]. A key contribution of this work is to quantify both, the magnitude and the time course of the ECM contraction around the spheroid and relate this to invasion. Hence, our study provides an insight into the dynamics of collagen contraction using the complex three-dimensional tumor model presented by the cancer spheroid. In contrast to single cell analysis we can identify a pushing event on the spheroid surface due to growth and a pulling event that dominates the collagen structure away from the spheroid, and that is originating from contractile forces. The described distinct phases of contraction (See Fig 3C and 3D) and their correlation with invasion shows that the contraction is preceding invasion, and that invasion will not stop when the contraction of the ECM stops. As expected, the contraction correlates with the accumulation of collagen at the surface of the spheroid. Furthermore, we show that tension is the cause of contraction and that the absence of tension inhibits invasion. We propose that tension is the relevant physical quantity for efficient invasion. This is in contrast to previous model that suggest the pure elastic modulus of the ECM as a key factor that influences invasion. In our cutting experiments the elastic modulus remains unchanged (as it is a material property) while the tension (load) is relaxed. Hence we propose tension as a new parameter that should be taken into account when analyzing invasion.

### Contraction phase Ia

In the initial phase, contraction starts immediately after seeding the spheroid and before the onset of invasion. Hence, invasion is not required for the contraction that has therefore to be initiated by the cells on the surface of the spheroid and the first internal layer of cells (as collagen penetrates the spheroid) and is not a result of contraction generated by the disseminated cells. Furthermore, this suggests that while cells are immediately able to apply traction forces, they cannot successfully invade in the first hours. Possible explanations for this delay may be intracellular adaptation, certain time required to degrade a first layer of collagen or the mechanical effect that the ECM is too soft to provide the mechanical resistance to sustain the forces required to detach a cell from the spheroid. We also observe over time the increase in the spheroid’s size and the development of characteristic alignment of collagen fibers (See Fig 2A). The tangential alignment of the collagen close to the spheroid and the radial alignment can be explained by a geometric effect that was shown previously for the alignment of collagen fibers between closely places fibroblast spheroids [7,32,45]. This model is simply applied to the compression zone at the spheroid surface and the dilation in the region where the contraction is observed (Fig 5D, blue sketched networks). The absence of radial alignment in previous studies on non-invading cells suggests that the active pulling is the relevant element for this organization. The radial alignment present in our model is not as pronounced as in *in vivo* reports. This can be explained by overall much higher complexity of *in vivo* stroma, where next to cancer cells contractile cancer associated fibroblasts are present that contribute to overall collagen organization.

Moreover, the accumulation of collagen parallel to the spheroid does not present a physical barrier for cells as they can still contract the distal collagen (Fig 2A).

The observed increase of contraction speed over time hints for an increase in applied forces, which may be due to an increase of forces generated per cell (e.g. mechanotransduction feedback [8]), or caused by an increasing number of cells (e.g. from the second or third cell layer) reaching outward, and thus increasing the total force applied on the collagen. Increasing matrix tension is known to trigger the maturation of focal adhesion [43] and activate expression of proteins regulating the cell contractile machinery [9] that might enhance the collagen contraction. Studies also showed that cells in 3D environment alter the contractility, which is observed by the change in shape in response to changes in mechanical loading to maintain tensional homeostasis [46]. From the delayed onset of invasion (9–12 h) we suspect that a certain level of mechanical remodeling and possibly biomechanical signaling needs to be developed before the invasion starts. A clear conclusion of this analysis is that cells exert forces on the environment long before migration starts.

### Slower acceleration in contraction phase Ib

During the second phase (12–30 h) the contraction area increases as cells move away from the spheroid and continue to invade. This phase is marked by a visible change in the slope of the curve in Fig 3C and lasts until the contraction speed reaches its peak at approximately 33 hours. The observed change of velocity retraction increase might have two plausible causes. Either the increase of forces applied from the cells starts to saturate or a potential onset of nonlinear stiffening might start to oppose the contractile stresses. Furthermore, the second phase starts with the onset of invasion, which raises the possibility of a contribution of the invading cells to the observed change.

The observed alignment might be a direct result of the observed collagen flows, such as described in *in vitro* systems under shear [30]. Besides direct pulling force of the cells, an elongation and reorientation of the collagen fibers on the surface by the cells might also explain the observed flows. Further detail studies on the relation between flow, collagen arrangement and fiber dynamics might help to understand this in detail. Additionally, the observed radial alignment at this later time-point could potentially emerge from cell penetrating the collagen, and hence arranging the collagen along their side [12]. This is however not very likely as the length scale of the collagen fibers is beyond single cells. Hence the gap created by the cells during movement is too small to rotate long fibers. Additionally, at the time point where the radial alignment is detected the cells are only starting to penetrate the region of radial alignment, excluding active remodeling by the cells as the origin for the reorientation. This suggests that the reason for the alignment is independent of the presence of cells at the location of radial alignment. Therefore we interpret the radial arrangement as a result of the radial collagen flow. Polymer flows are knows to align individual filaments. In this view the concentric alignment is due to the condensation on the spheroids surface, while in the larger distance the radial puling forces of the cells of the spheroid result in the radial alignment.

### Deceleration in contraction phase II

In our experiments we have an average accumulated displacement of about 14 μm at the beginning of the third phase. Combined with an estimated length scale of 300 μm at which no contraction was observed we enter a regime of 5% deformation [26,47]. It should be noted however, that the reported values of the total deformation corresponds to the average within a 100 μm rim around the spheroid surface, thus underestimating the total deformation at the surface of the spheroid. Collagen networks are known to show nonlinear strain stiffening already at 10% deformation. These non-linear effects are commonly explained by geometric realignment of the network [47], and hence we suspect that the found alignment of the fibers indicates that the collagen is in a non-linear stiffening regime. This effect would explain why the displacement velocity gradually decreases in phase II, because the collagen network stiffens. As the invasion continues during this phase, the reduced contraction is not due to a release of force applied by invading cells.

### Cutting the collagen reduces tension built up and invasion

Literature suggests matrix tension as an important factor driving tumor progression [9]. Previous studies in non-cancerous cells showed that migration patterns are associated with matrix tension lines [31–33,35]. The presented cutting experiments show that even in the absence of ongoing deformation, the ECM still bears mechanical tension and that possible viscoelastic relaxation on the timescale of days is not sufficient to fully relax tension.

There are several possibilities of what types of matrix properties need to be changed in order to support invasion. Previous work showed that a tumor’s matrix has characteristic geometrical and morphological features such as parallel fiber alignment and specific pore size. This alignment can then provide structures that act as highways for invading cells [14,39,40]. In this model cells need to first align collagen before they can invade most efficiently. While we find an alignment of the collagen due to the contraction, our cutting experiments suggest that the actual increase of tension forces in the ECM trigger invasion rather than the geometrical changes that are caused by the contraction. In the direction of the cut, we have a strong contraction due to the absence of mechanical support. However, the invasion is strongly reduced. Moreover, on the opposite of the cut side, the contraction was not increased but invasion was still affected. From a force equilibrium point of view, we know that the total tension over the spheroid’s surface has to cancel. Any net force would result in the spontaneous movement of the spheroid. Hence, the tension experienced by the cells located on the side facing the cut and on the side opposing the cut is similar. Note the contraction itself facilitates the invasion but the presence of tension in the ECM seems to be the critical mechanical parameter that triggers or facilitates invasion. The absence of such tension in direction towards and opposing the cut might be therefore the reason for the decreased invasion in these directions. Another interpretation of these experiments is that the cells may not be able to move out because the reference system of the cells, presented by the collagen gel, is moving inward rapidly. However, the definition of invasion is the process of detachment of cells from the tumor, and not the relative speed in the reference frame. They pull on the collagen simply moving the network towards the spheroid with the same speed they could migrate in a fixed environment, but in the absence of tension the cells cannot leave the spheroid.

Additionally, when the cut is performed 24 hours post-seeding when cells already established fiber alignment and contracted the gel, the disruption of the tension still resulted in inhibition of invasion (S6 Fig), showing that the tension is the defining factor and not only fiber alignment. In contrast, we show that on the area of the gel where tension on fibers was expected to be highest (perpendicular to the cut) we observe increased invasion. This experimental result provides direct support for a tension based model for cell invasion [34,43].

Previous work argues that the cells sense the ECM mechanics and require that a certain mechanical stiffness (elastic modulus) is met [48,49]. The macroscopic cutting experiments were performed to separate the effect of elastic modulus from the tension. The elastic modulus is a material property and it does not change when the material is cut. The cutting of the gel was done right after gel was polymerized; hence elastic modulus at time 0h was equal at the side of the cut and all other sides. No other modification to gel was applied.

### Tension-driven invasion model

Summing up our observations we propose a model of invasion based on self-remodeling of the 3D matrix by the CT26 cancer cells spheroid (Fig 5D). After seeding the spheroid in randomly dispersed collagen, the first layer of cells establishes a connection with fibers using adhesion proteins. They sense the rigidity, tension and fiber alignment and trigger specific biochemical signaling that remodel the matrix further. Besides this biochemical remodeling, cells start to generate traction forces and contract the collagen radially around the spheroid (black arrows). As result of this radial contraction, fibers on the surface align parallel to the spheroid, while the fibers in the more distal parts align radial and come under tension (white arrows). This is shown in the simplified ECM geometry at the top of the sketch in blue. The growing spheroid pushes collagen fibers on the surface thus leading to further increase of collagen density and parallel alignment in the first collagen layer (blue arrows). Besides these more steric reasons for the parallel collagen alignment, both biochemical remodeling and forced rearrangement due to tangential forces are possible explanations. Cells form radial protrusions that contract the outer collagen, hence increasing the mechanical tension that is applied by the cells and transmitted to their neighbors. Once a critical tension is reached, the cell-cell contract ruptures and the cells can leave the spheroid. The invading cells align then along the radial fibers and migrate away from the spheroid.

Cells from the underlying layers follow and further apply traction forces resulting in an increase of tension, which eventually leads to strain stiffening of the collagen and a decrease of contraction.

When the collagen fibers are cut and a free surface is created, the cells in the direction of the cut are not able to generate sufficient tension in the ECM to detach from the spheroid. Additionally, force-sensitive intracellular signaling might not be triggered in this situation.

In this model the alignment of fibers is explained as a simple geometrical feature of the spheroids expansion and the collagen contraction. The simultaneous onset of invasion is then a result of force balance that predicts an equilibration of tension over the spheroid and a plausible critical tension that is determined by the cell-cell adhesion strength. The radial outgrowth is explained by the anisotropic tension and the radial alignment of the fibers. Finally, the decreased invasion upon tension release is a direct prediction of the model.

## Conclusion

While the biochemical changes observed in tumor invasion are undoubtedly important features, we here highlight pure physical effects that are the result of cells actively pulling on a collagen matrix. If the matrix does not support invasion, these pulling forces do contract the surrounding ECM and thereby auto-organize both, the geometrical structure and the tension carried by the ECM. This, in turn modifies the ECM properties until invasion is possible. We conclude that the tumor can by a simple force application optimize the mechanical properties, eventually leading to an invasion of the cells into the surrounding environment. A requirement for this model is that invasive cells apply forces to the environment.

## Materials and Methods

### Cell culture and preparation of multicellular cancer spheroids

Mouse colon cancer CT26 cells (Cat. No. CRL-2638; American Tissue Culture Collection), that are stably transfected to express cytoplasmic GFP are cultured in DMEM medium supplemented with 10% (v/v) fetal bovine serum (FBS), 2% (v/v) antibiotics (100 μg·mL−1 streptomycin and 100 U·mL−1 penicillin) (Life Technologies). Cells are maintained at 37°C in a 5% CO_2_ humidified atmosphere. Cells are passaged every 2 days to avoid reaching confluence that might cause cell differentiation.

Multicellular cancer cell spheroids are prepared from CT26 cells using a classical agarose cushion protocol (S1A Fig) [50]. Briefly, agarose (Life Technologies) is boiled in water (sterile) to achieve a 1% solution. 150 μl of this solution is immediately added to each of a 48-well plate under a laminar flow hood. The plate is cooled down to room temperature before adding cells. Cells are used at sub-confluence, detached from the flask with trypsin (Life Technologies) and counted. Cell suspensions are prepared and added to each well of the plate to achieve cell concentrations corresponding to 2000 or 1000 cells per well. Plates containing cells were incubated for three days at 37°C and 5% CO_2_, humidified air.

### Labeling of collagen type I with TAMRA

Labeling of collagen type I with TAMRA is performed following method described previously [25]. Briefly, rat tail collagen I (non-pepsinized; BD Biosciences, Cat. No. 354236) is dissolved in 0.2% (v/v) acetic acid and dialyzed overnight with labeling buffer (0.25 M NaHCO3, 0.4 M NaCl, pH 9.5) at 4°C. Fluorophore tetramethyl- rhodamine (TAMRA, Invitrogen) is resuspended in DMSO according to the manufacturer’s instructions and diluted in labeling buffer. Collagen solution and TAMRA is mixed and incubated overnight at 4°C under agitation. Free dye is removed by dialyzing the labeled collagen against labeling buffer overnight at 4°C. TAMRA-labeled collagen is finally dialyzed overnight against acetic acid 0.2% (v/v) at 4°C.

### Three-dimensional collagen assay

In this study, a restrained matrix contraction model is used [35]. Based on the experimental results that the contraction in the far field is not measurable, we consider the approximation of an infinite collagen gel. As the introduction of walls close to the collagen might have led to additional orientation effects and further complexity to the subject of the study, the gel was exclusively attached to the bottom.

S1B Fig shows the subsequent steps of 3D collagen assay preparation. The fluorodish chambers (World Precision Instruments, Cat. No. FD35-100) are coated with 50 μg/ml collagen type I (diluted in 0.2 N acetic acid) for 1 hour at room temperature to coat the glass surface to provide a better attachment for the polymerized gel later. After incubation with collagen the dish is washed with PBS and air-dried. Dishes are stored at 2–8°C for up to one week under sterile conditions.

Collagen polymerization mix is prepared to yield a final concentration of 2 mg/ml collagen solution. Polymerization mix is prepared by mixing unlabeled rat tail collagen type I, filter-sterile 10 x PBS, 1N NaOH and DMEM or Leibovitz’s Media (for life-imaging) under sterile conditions on ice. To visualize collagen fibers, TAMRA-labeled collagen type I is introduced into the mix in proportion 1:6 to the unlabeled collagen. For some experiments, collagen gel is co-polymerized with red (580/605) fluorescent carboxylated beads (4μm diameter, FluoSpheres Sulfate Microspheres, Cat. No. F-8858, Life Technologies) at 5% v/v. Polymerization mix is mixed well and the pH of the final solution is tested with a pH test strip to ensure pH 7.5. A 20 μl drop of collagen solution is pipetted onto the collagen-coated glass bottom dish. A spheroid is aspirated and placed on a clean Petri dish. Excess of liquid is removed to prevent dilution of the collagen with cell media and 10 μl of this unpolymerized collagen mix is added on top of the spheroid. The collagen-suspended spheroid is aspirated and placed on the bottom of the 20 μl collagen mix drop immediately after neutralization of the collagen’s pH. The collagen mix with inserted spheroid is polymerized at RT for 1 hour. The dish is flipped up and down several times to place spheroid in the middle of the collagen drop. When polymerization is finished, 2 ml of culture medium is added to cover the collagen/spheroids drops. Dishes with collagen/spheroid are placed on the microscope equipped with a stage incubator at 37°C for live imaging.

### Fluorescent and pseudo-speckle microscopy and basic image corrections

To analyze the collagen contractile flow, we use a technique called pseudo-speckle microscopy, which is conceptually similar to particle image velocimetry (PIV). The technique is also related to fluorescent speckle microscopy (FSM). FSM provides images with bright fluorescent speckles on a dark background that are then tracked over time. As opposed to this technique in our approach we use bright-field images of collagen, and we exploit the optical inhomogeneities of the sample, to which we refer to as pseudo-speckles [41,51,52]. For the deformation analysis, samples are imaged using a Nikon Inverted Research Microscope (ECLIPSE Ti), a Spinning Disk module (Yokogawa CSU-X1), an xy-stage (ASI PZ-3500) equipped with a piezo stage (Mad City Labs Nano-Z500) and a sCMOS camera (Andor Neo). A CFI S Fluor 4x (0.2 Numerical Aperture NA), a 10x (0.45 NA) and a 20x (0.75 NA) objective are used to image the cells. The microscope is controlled by the IQ2 software (Andor). High-resolution images (1040 x 1390 pixels) are acquired in bright field, GFP-positive cells are imaged using a 491 nm laser, TAMRA-labeled collagen fibers and red beads are imaged using 561 nm laser. The laser is typically used at 10–15% power. 40 μm z-stacks (5 μm slice separation) of spheroids are acquired every 1–3 hours. To analyze collagen contractile flow, a technique equivalent to PIV is used. Bright field images of collagen are acquired as described above and the optical inhomogeneities of the sample are used to determine the collagen contraction [51]. The bright field images are combined with fluorescent images of GFP-positive cells and collected on a spinning disk microscope.

Images are saved in a 8-bit TIFF format. “Maximal Intensity” projection along the z-axis is created using the ImageJ software. In the case of bright field images, only the middle slice of the Z-stack is analyzed. Both bright field and fluorescent images are corrected for drift using the “StackReg” and for photobleaching using “Bleach Correction” functions of ImageJ. To facilitate edge detection of the spheroids, contrast and brightness are corrected (contrast level should be relatively high). The diameter of spheroids is measured using ImageJ.

### Cell invasion quantification

Tumor cell invasion is defined as translocation of neoplastic cells through host cellular and ECM barriers [53], which indicates that cells must detach fully from the tumor. We based our invasion analysis on this definition. The method for quantification of invasion used in this paper has been previously published [41].

All operations are done in ImageJ. S2 Fig shows steps of image manipulation to quantify invasion. Fluorescent images of GFP-positive cells are z- projections as described above. Images are binarized using “Huang” filter. The “Analyze particles” function is used to acquire the outline of the spheroid and invading cells. Only the particles detached from the core (invasion area) are taken into account for quantifications. The area and radius of the spheroid at time 0h (total area) is also calculated using the “Analyze particles” function. Each specific invasion area is normalized to the total area of this spheroid at time 0. “Radial profiles” and kymographs are acquired using ImageJ.

### Collagen deformation analysis

Collagen pseudospeckle movement is analyzed with in-house developed tracking software simiar to PIV [54,55]. The software is written in the Matlab programming environment (The MathWorks). Bright field images of collagen and spheroids are used in a png format to analyze collagen movement. The spheroid and the invading cells’ area are removed from the contraction analysis, using the edge detection function of the program. The analysis of collagen movement is based on a cross-correlation between the initial and the following image. Two subsequent images are separated into small sub-images, and a maximum correlation algorithm is used to determine the displacement of prominent parts from one image to the next one. The method is visualized in S7 Fig. As the cells contract the collagen, the dark particle (marked by the white arrow) moves towards the growing spheroid. The images are divided into a grid. A sub-image called “Source” is cut and around the same grid-point, but in the subsequent image, a second sub-image called “Search” image is cut. A correlation map is constructed, where the source image is compared to the search image by calculating the cross-correlation value between the source and all possible sub-images of the search image that correspond to the size of the source image. The color and gray scale maps of deformation velocities are generated. These maps are interpolations from the values determined on the grid-map created in the previous step. Arrows indicate the direction of collagen movement while color indicates the speed according to the generated scale bar. For quantification of average pixel intensities of the contraction area surrounding the spheroid, gray scale deformation maps are used. The spheroid pixel area is subtracted from the analysis using ImageJ as follows. Z projected fluorescent images of GFP-positive cells are used to segment spheroids with ImageJ. Images are binarised using “Huang” filter. “Analyze particles” function is used to acquire the outline of the spheroid. Only the biggest particle (core of spheroid), and not the detached from core particles (invading cells) are taken into account. The binary map of spheroid is applied on the gray scale images of the deformation field. On each image 70 pix radius (approximately 100 μm) area is selected and mean gray intensity for each time point is measured. The value of 255 corresponded to the maximal deformation velocity that is chosen for a given data set.

Collagen orientation was measured using “OrientationJ” plugin of ImageJ [56].

### Microsurgery of collagen fiber network

Spheroids are seeded into unlabeled collagen gels following the procedure described above. Spheroids are left in collagen for 48 hours before ablation experiment. Control gels are polymerized under the same conditions but without the spheroid. Laser ablation and imaging are performed using an inverted LSM710 (CarlZeiss) microscope equipped with an incubation chamber (Pecon) maintaining the 5% CO_2_, 37°C temperature and humidity. GFP-positive cells are imaged using a 488-nm (25-mW) argon laser and a 25x /0.80-NA or 40x /1,3-NA oil immersion objective. Collagen fibers are imaged using the reflection mode with the same 488-nm laser. Ablation is performed using a 2-photon laser system based on the Mai-Tai HP laser (Spectra Physics) at 800 nm with pulse width < 100 femtoseconds. The area of ablation is a square between 10–20 μm (x-y). Images are taken before and after ablation every 10 seconds. Z projections of 10 μm stacks (taken every 1 μm) are used for the image analysis and to perform kymographs. The movement of fibers after ablation is manually tracked and the x-y position is recorded.

To estimate the tension, we manually track the relaxation of the collagen around the cut. The relaxation profile presented in Fig 4 shows first a fast relaxation of the tension, followed by an exponential relaxation that is slowed down by the intrinsic viscosity of the system. In this work, we are only interested in estimating the tensile forces. For this a quantitative measure of the strain is required (see Fig 4A), which is defined as the recoil at the site of cut (L_d_) divided by the distance at which no movement of collagen was visible (L_0_).

### Macrosurgery of collagen fiber network

Spheroids are seeded into collagen type I either TAMRA-labeled or unlabeled with addition of 4 μm red fluorescent beads (5% v/v) as described above. Gels are polymerized as described above and then either immediately cut or incubated for 24 hours at 37°C and 5% CO_2_ (S4 Fig). The cutting of gels is performed under stereoscope Leica MZ10F with PLAN APO 1x objective (Leica Microsystems) using sterile scalpel (Swann-Morton). The cut is done at a distance of 100–300 μm from the spheroid. Dishes with collagen/spheroid are placed on the Nikon spinning disk microscope equipped with a stage incubator at 37°C for live imaging. Area of invasion is calculated using method described above on the cut side of the gel and on the uncut side in ImageJ. Briefly, first the line perpendicular to the cut is drawn between center of the spheroid and edge of the gel. Using the “angle” tool the area of 45° on each side of the line is selected. The invasion area is measured in this selection. The remaining area of invasion is also measured and divided by three to represent average invasion on the uncut side of the gel. “Polar transformer” plugin of ImageJ (Polar 360 degree transform) is used to convert images to polar coordinates and “unwrap” spheroids and invasion zone. 300 pixels in width line were drawn across the unwrapped spheroid and profile fluorescence was measured using the “Plot profile” tool.

### Intravital imaging

Intravital imaging of intestinal tumors is performed on NICD/p53-/- mice [57]. In this mouse model, the formation of invasive carcinoma is driven by the expression of a conditionally activated Notch1 receptor (NICD) and the deletion of the p53 tumor suppressor in the digestive epithelium. All intestinal epithelial cells express nuclear GFP.

Intravital imaging was performed on an upright Nikon microscope (A1R-MP) with an Infrared laser (Insight Deep See, Spectra). Cancer cells were visualized by their nuclear GFP signal collected on a GaASP detector with a BP 550/25 filter. Collagen structure is imaged using Second Harmonic Generation (SHG), on a GaASP detector with a SP 492 filter. The sample was excited at 960nm.

## Supporting Information

S1 Fig Methodology of the 3D invasion assay. **(A)** Sketch of the CT26 spheroid generation agarose method. **(B)** Sketch of collagen 3D invasion assay. **(C)** Comparison of collagen type I morphology polymerized at room temperature and 37°C. Scale bar: 20 μm.
(TIF)

S2 Fig Method of quantification of invasion area. GFP-positive spheroid z projection images are binarised and “Analyze particles” function of ImageJ is used to acquire detached particles area.
(TIF)

S3 Fig Collagen contraction dynamics in phase 2. In some cases the contraction in phase 2 did even plateau to allow a clear and direct visual identification of the three different phases. The errors are the STD of the measured retraction in the 100μm rim of this particular experiment.
(TIF)

S4 Fig Tension-related cell invasion pattern. **(A)** Invasion of two CT26 GFP spheroids (green) in collagen (red, imaged in reflection mode) showing cells lining up in straight lines directly connecting the two spheroids. Image is taken 48 hours post-seeding in collagen. **(B)** Magnification. Scale bar: 200 μm.
(TIF)

S5 Fig Sketch of gel macrosurgery experiments. Red line indicates the area where gel is attached to the surface of the dish.
(TIF)

S6 Fig Direction dependent invasion during collagen gel macrosurgery. When macrosurgery is performed after 24h when the cells have already stated to invade the spheroid. At 0h and 24h (right before cut), no dependence of the outgrowth on the direction is observed. After 72h a decrease in the direction is seen, however this is less pronounced as in the case of cuts performed immediately after polymerization (main text Fig 5A and 5B). The shading of the graph shows the side facing the cut (light blue), the opposing side facing the inside of the gel (light red) and the two sides perpendicular to the cut (white area).
(TIF)

S7 Fig Method of collagen contraction quantification. A cross-correlation method is used by dividing the image into a template pixel area called Source (green square). The Source is overlaid onto each pixel of a larger area “Search” (violet square) at the same grid point in the successive image. The cross-correlation value for each Search pixel is calculated and represented by color map and arrows. Scale bar: 50 μm.
(TIF)

S8 Fig Overlay of the deformation detection obtained from particle tracking and PIV. (TIF)

S9 Fig Correlation of between the deformation detection via particle tracking and PIV. On the same dataset both methods were used. The particle tracking is based on fluorescent beads embedded in the collagen. To compare the detection quantitatively for each frame the displacement of the bead was compared to the measured displacement from the PIV. The x-axis shows the deformation of detected via particle tracking, and the y-axis shows the corresponding deformation detected via PIV. Only integral steps are shown in the x-axis as the particle tracking has pixel-based resolution. Overall high correlation is obtained, with only small deviations for large displacement, where the smoothing effect of the PIV starts to influence the data.
(TIF)

S1 Movie Projection of TAMRA-labeled collagen type one polymerized at room temperature. Projection of 30 μm (slices every 1 μm). Scale bar: 10 μm.
(MOV)

S2 Movie Invasion of CT26 into a three-dimensional collagen gel. Images are recorded every 1 hour for 24 hours. Z projection of 100 μm. Left: Bright field. Green: CT26-GFP cells. Red: Collagen labeled with TAMRA. (D) Merged GFP cells and collagen.
(AVI)

S3 Movie Comparison of movement during collagen contraction by CT26 spheroids. Bright field (left) and fluorescent beads (right). Beads images present the z projection of 40 μm stack.
(AVI)

S4 Movie Validation of collagen deformation analysis (left) using commercial software bead tracking tool (right, Imaris). (AVI)

S5 Movie Dynamics of the deformation analysis. Overlay of the deformation detection obtained from particle tracking and PIV.
(MP4)

S6 Movie Ablation of collagen fibers. Collagen is cut using 800 nm laser and imaged by reflection mode (at 488 nm) every 10 seconds. Separate images are a z projection of a 3 μm stack.
(AVI)

S7 Movie Invasion of GFP-CT26 after cutting of collagen gel on one side. Note the movement of the cut line towards the spheroid (red line). Separate frames are z projection of 50 μm stack.
(AVI)
