# Supplementary figures and images for: Tensile Forces Originating from Cancer Spheroids Facilitate Tumor Invasion
Source: PLoS One. 2016 Jun 7;11(6):e0156442. doi: 10.1371/journal.pone.0156442 (PMC4896628; doi:10.1371/journal.pone.0156442)

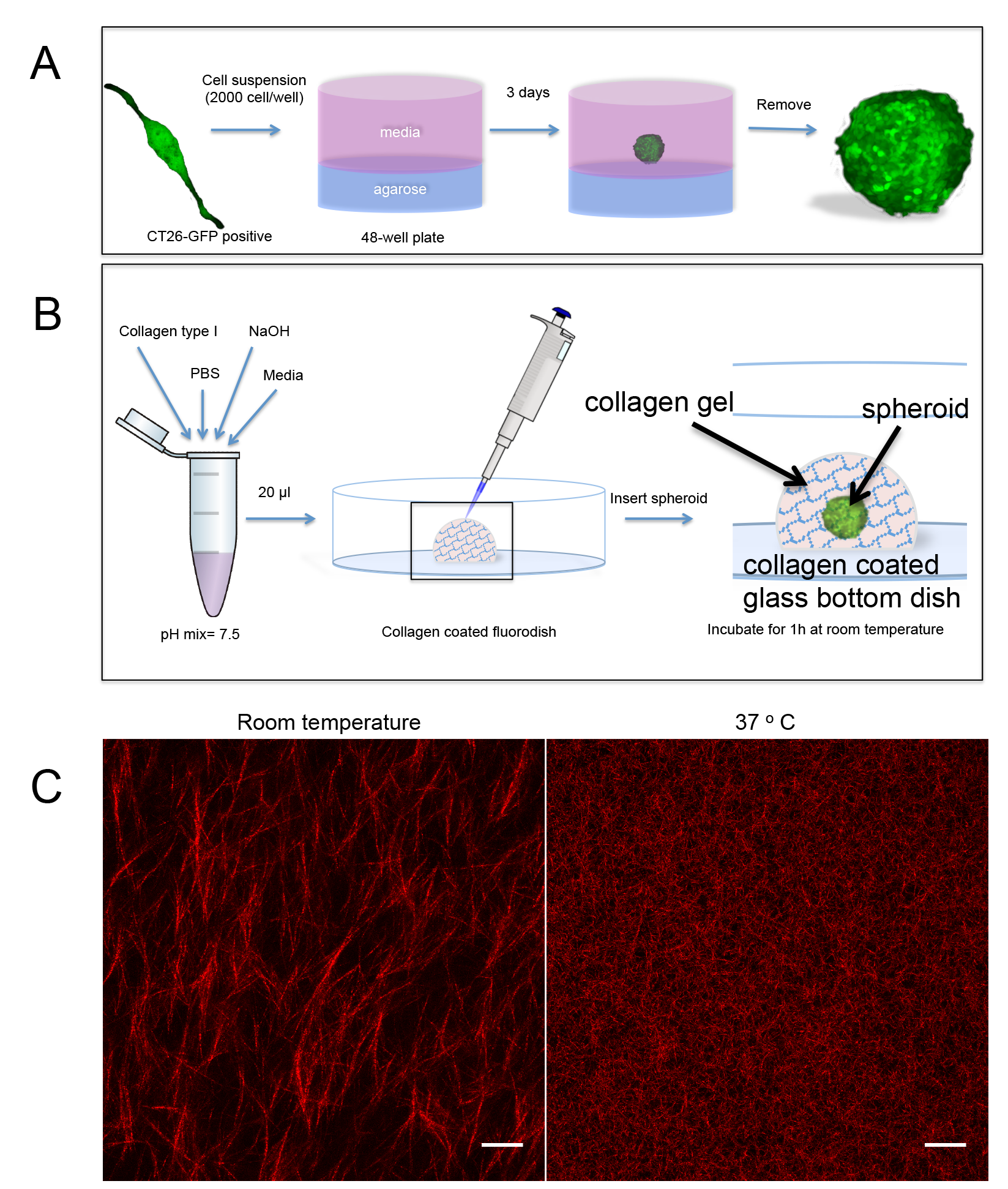

Supplement: S1 Fig — (A) Sketch of the CT26 spheroid generation agarose method. (B) Sketch of collagen 3D invasion assay. (C) Comparison of collagen type I morphology polymerized at room temperature and 37°C. Scale bar: 20 μm. (TIF) [file pone.0156442.s001.tif]

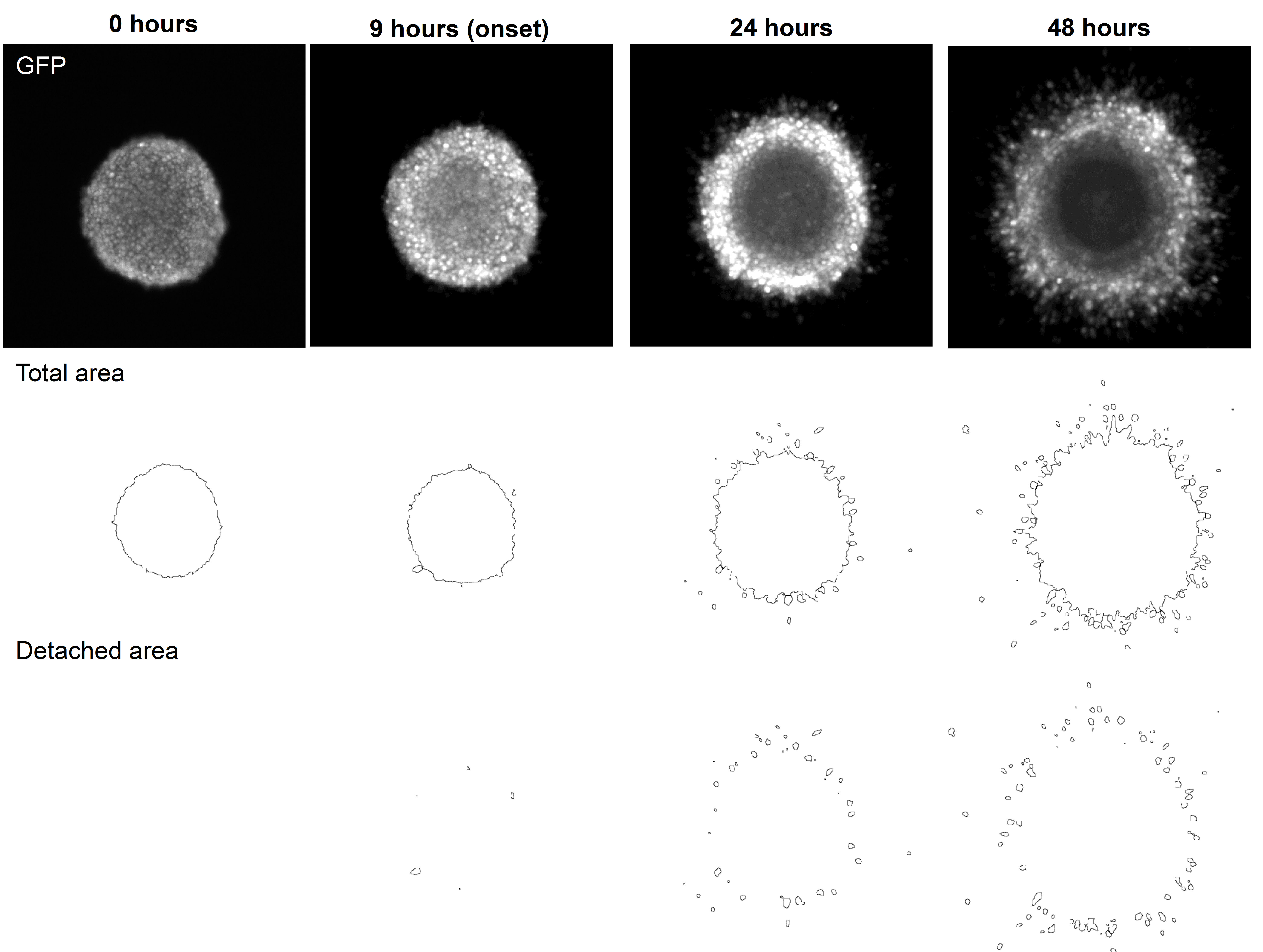

Supplement: S2 Fig — GFP-positive spheroid z projection images are binarised and “Analyze particles” function of ImageJ is used to acquire detached particles area. (TIF) [file pone.0156442.s002.tif]

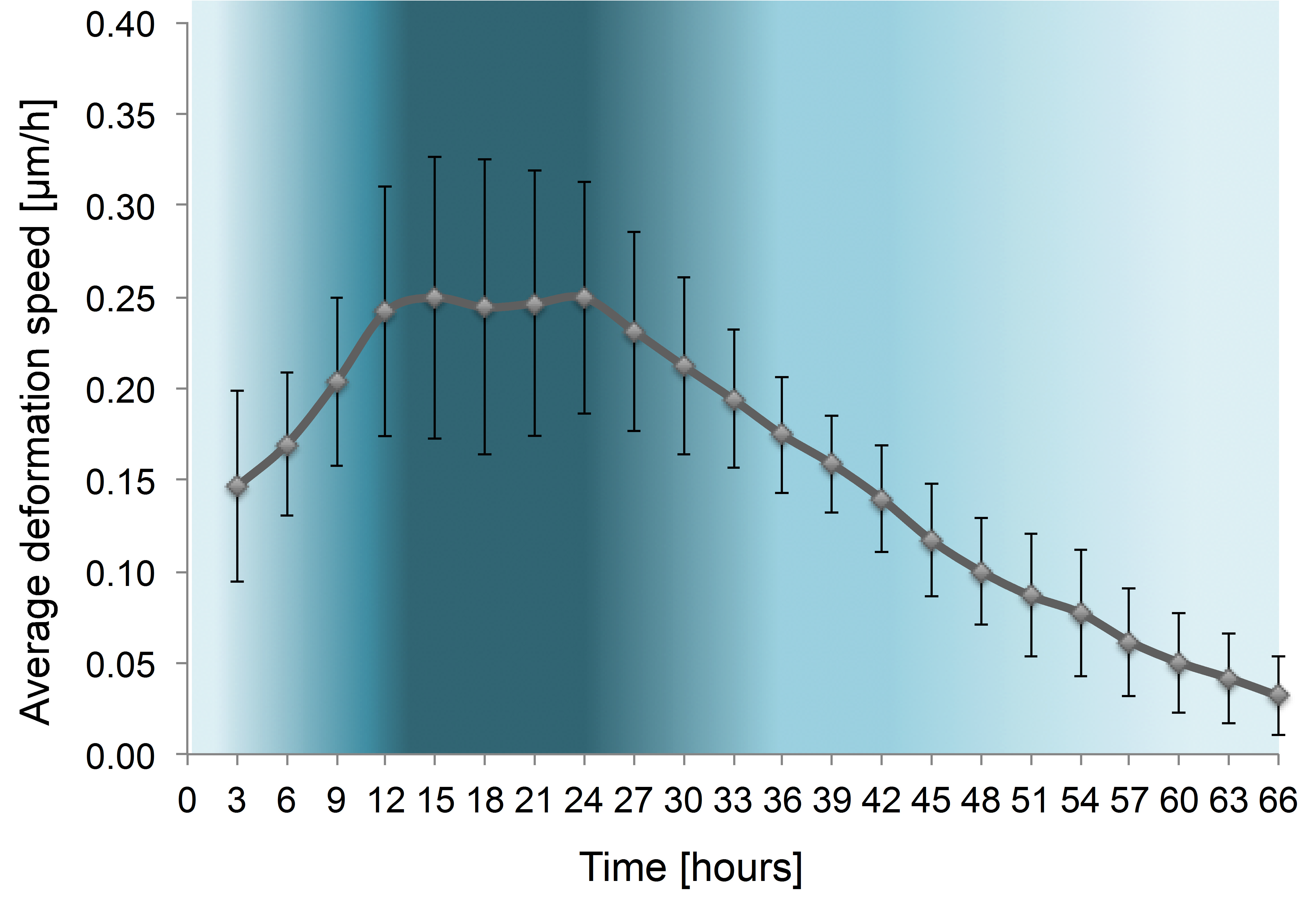

Supplement: S3 Fig — In some cases the contraction in phase 2 did even plateau to allow a clear and direct visual identification of the three different phases. The errors are the STD of the measured retraction in the 100μm rim of this particular experiment. (TIF) [file pone.0156442.s003.tif]

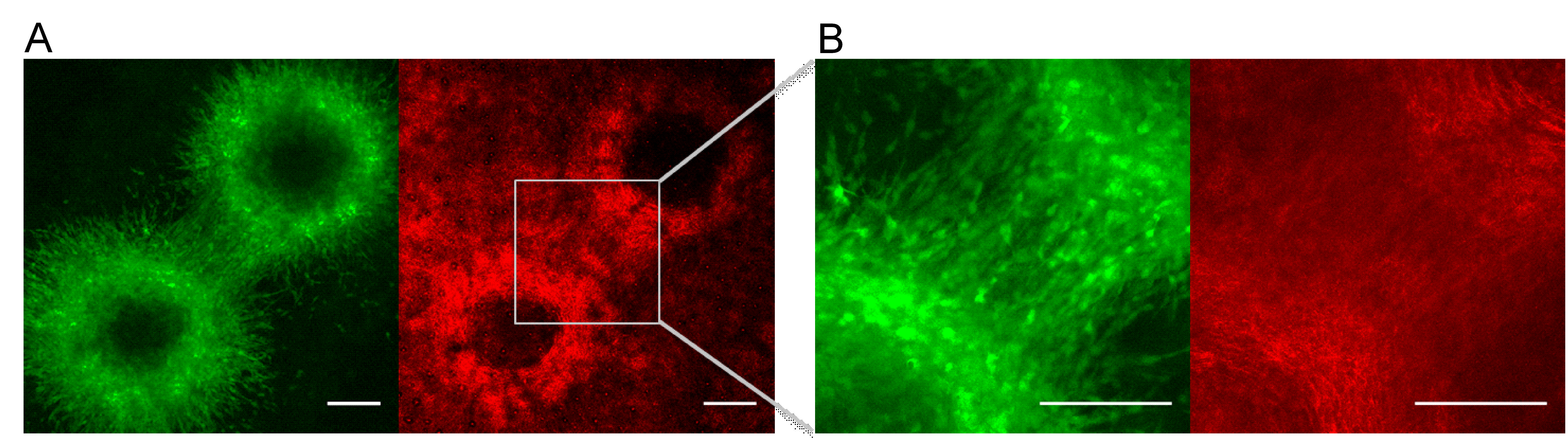

Supplement: S4 Fig — (A) Invasion of two CT26 GFP spheroids (green) in collagen (red, imaged in reflection mode) showing cells lining up in straight lines directly connecting the two spheroids. Image is taken 48 hours post-seeding in collagen. (B) Magnification. Scale bar: 200 μm. (TIF) [file pone.0156442.s004.tif]

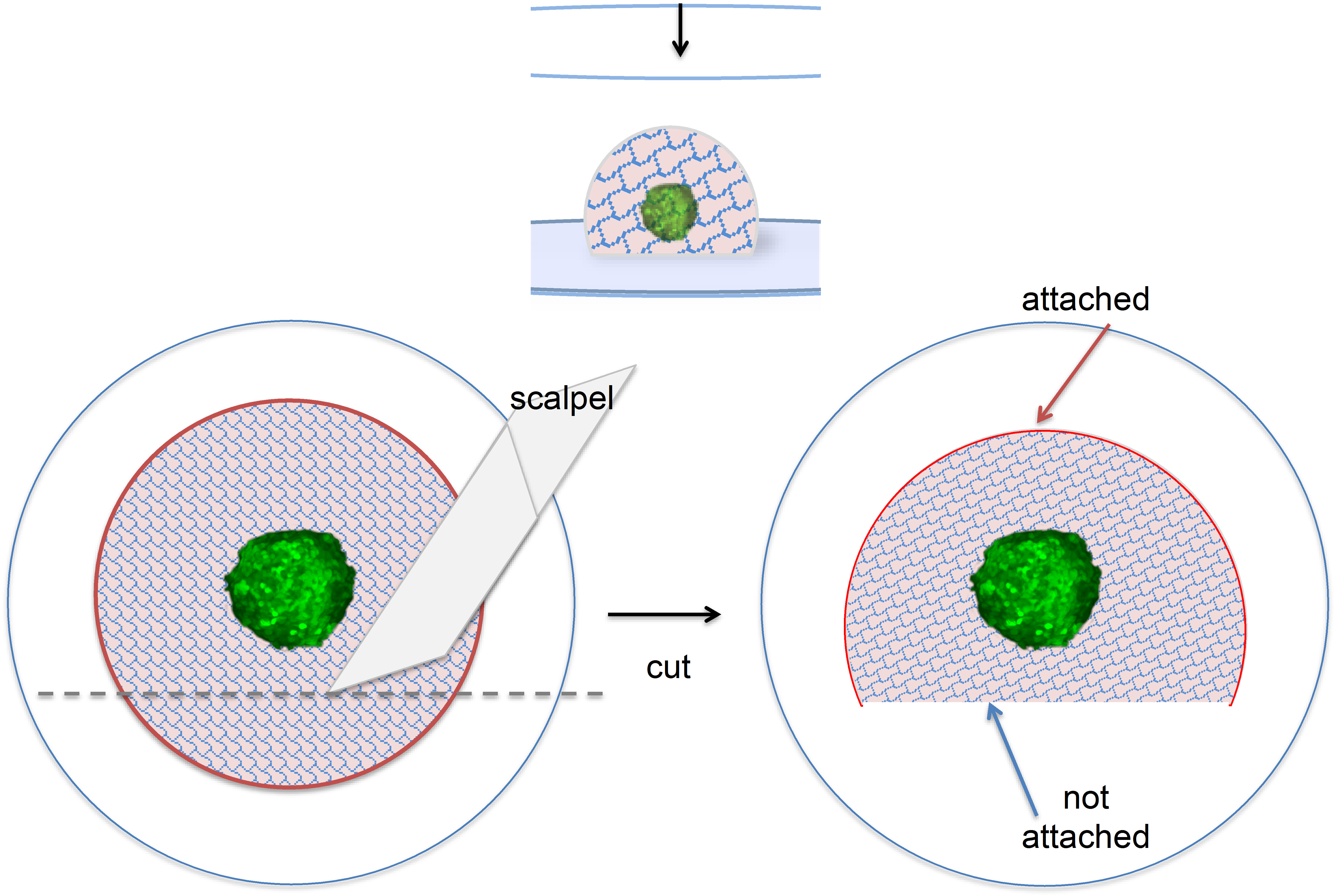

Supplement: S5 Fig — Red line indicates the area where gel is attached to the surface of the dish. (TIF) [file pone.0156442.s005.tif]

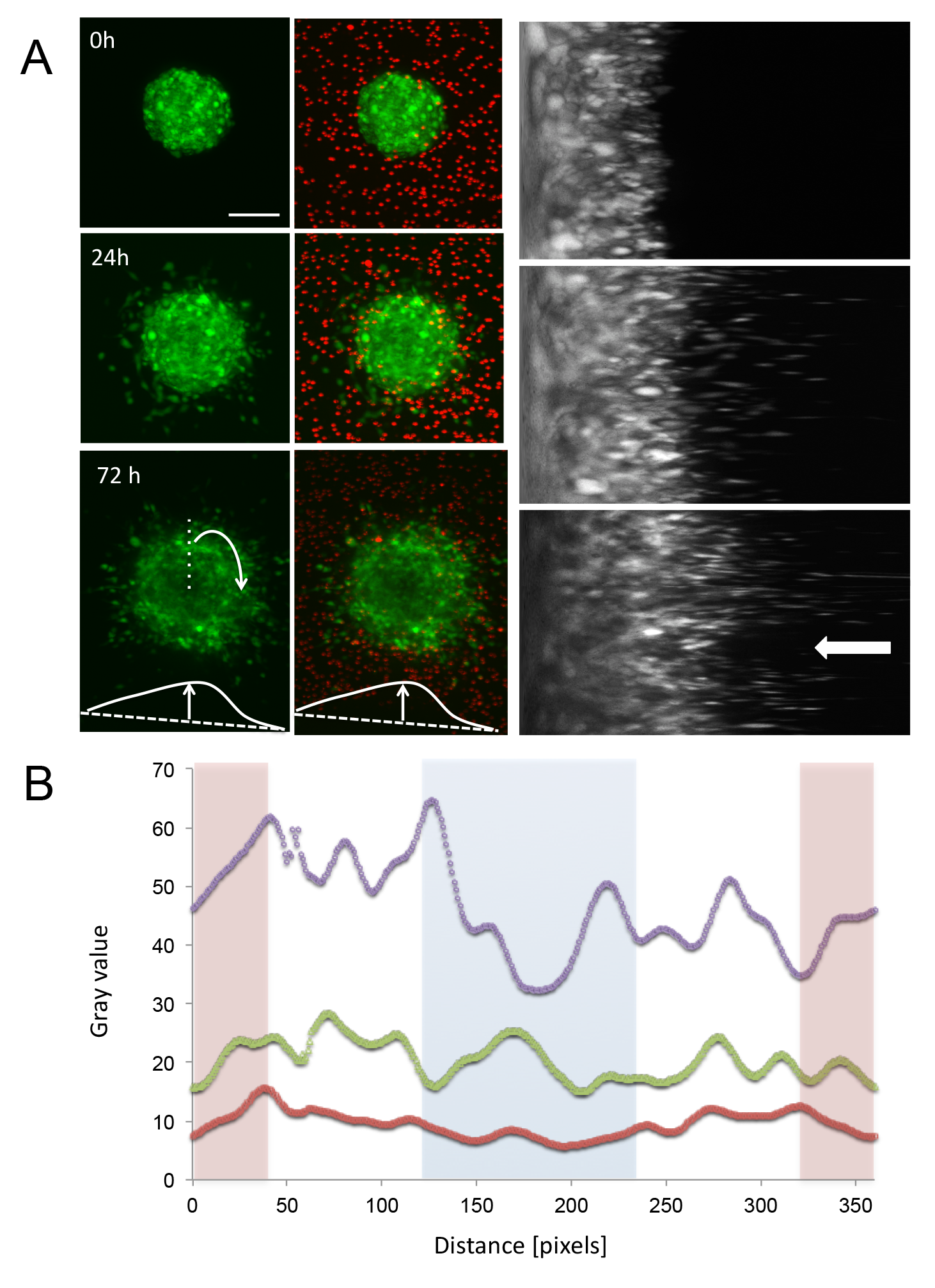

Supplement: S6 Fig — When macrosurgery is performed after 24h when the cells have already stated to invade the spheroid. At 0h and 24h (right before cut), no dependence of the outgrowth on the direction is observed. After 72h a decrease in the direction is seen, however this is less pronounced as in the case of cuts performed immediately after polymerization (main text Fig 5A and 5B). The shading of the graph shows the side facing the cut (light blue), the opposing side facing the inside of the gel (light red) and the two sides perpendicular to the cut (white area). (TIF) [file pone.0156442.s006.tif]

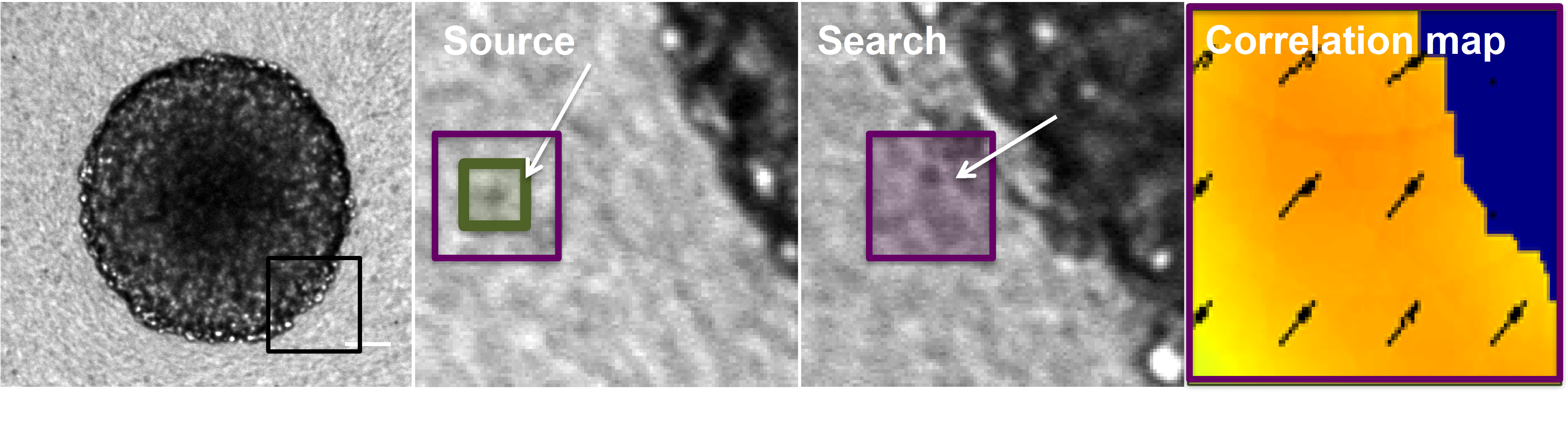

Supplement: S7 Fig — A cross-correlation method is used by dividing the image into a template pixel area called Source (green square). The Source is overlaid onto each pixel of a larger area “Search” (violet square) at the same grid point in the successive image. The cross-correlation value for each Search pixel is calculated and represented by color map and arrows. Scale bar: 50 μm. (TIF) [file pone.0156442.s007.tif]

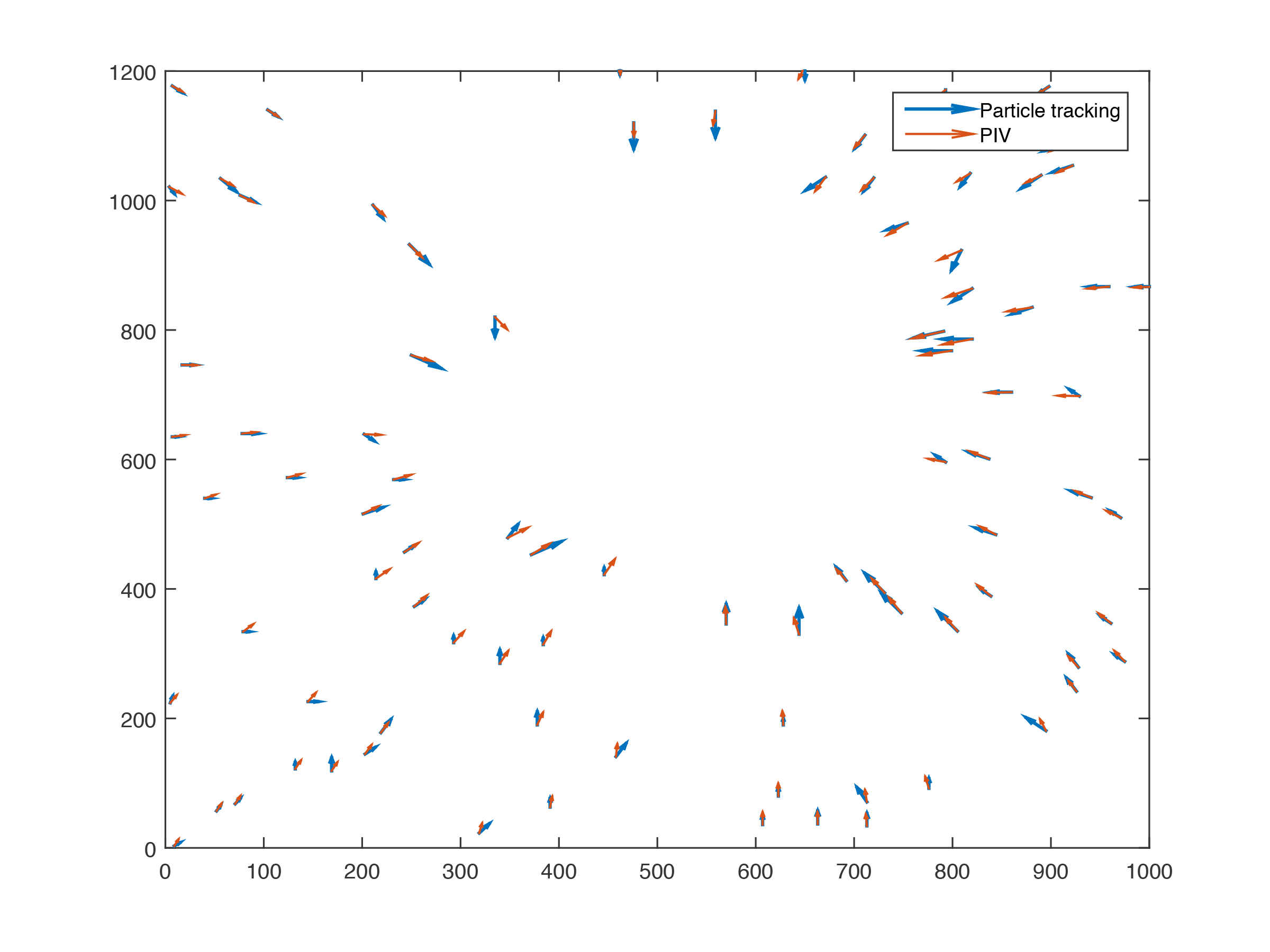

Supplement: S8 Fig — (TIF) [file pone.0156442.s008.tif]

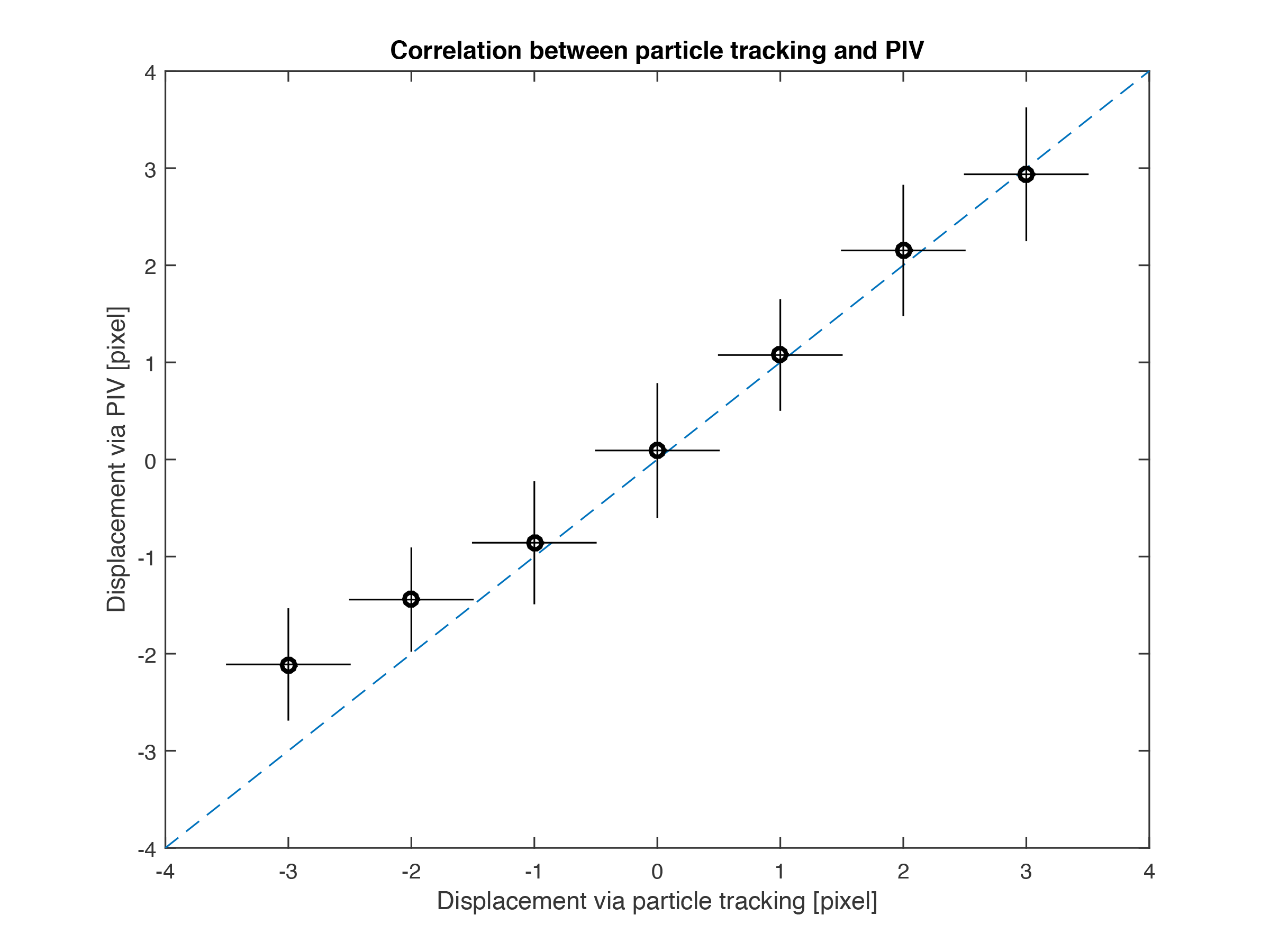

Supplement: S9 Fig — On the same dataset both methods were used. The particle tracking is based on fluorescent beads embedded in the collagen. To compare the detection quantitatively for each frame the displacement of the bead was compared to the measured displacement from the PIV. The x-axis shows the deformation of detected via particle tracking, and the y-axis shows the corresponding deformation detected via PIV. Only integral steps are shown in the x-axis as the particle tracking has pixel-based resolution. Overall high correlation is obtained, with only small deviations for large displacement, where the smoothing effect of the PIV starts to influence the data. (TIF) [file pone.0156442.s009.tif]
